# Epidemiological and genetic characteristics of clinical carbapenem-resistant *Acinetobacter baumannii* strains collected countrywide from hospital intensive care units (ICUs) in China

**DOI:** 10.1080/22221751.2022.2093134

**Published:** 2022-07-04

**Authors:** Congcong Liu, Kaichao Chen, Yuchen Wu, Ling Huang, Yinfei Fang, Jiayue Lu, Yu Zeng, Miaomiao Xie, Edward Wai Chi Chan, Sheng Chen, Rong Zhang

**Affiliations:** a School of Medicine, Department of Clinical Laboratory, Second Affiliated Hospital of Zhejiang University, Hangzhou, People’s Republic of China; b Department of Infectious Diseases and Public Health, Jockey Club College of Veterinary Medicine and Life Sciences, City University of Hong Kong, Kowloon, Hong Kong; c Department of Clinical Laboratory Medicine, The Women’s and Children’s Hospital of Linping District, Hangzhou, People’s Republic of China; d Department of Clinical Laboratory, Jinhua Municipal Central Hospital, Jinhua, People’s Republic of China; e State Key Lab of Chemical Biology and Drug Discovery, Department of Applied Biology and Chemical Technology, The Hong Kong Polytechnic University, Hung Hom, People’s Republic of China

**Keywords:** *Acinetobacter baumannii*, carbapenem resistance, OXA-23, clonal transmission, molecular epidemiology

## Abstract

*Acinetobacter baumannii* is one of the key Gram-negative pathogens that can cause serious nosocomial infections. In China, a large proportion of clinical *A. baumannii* strains are multidrug resistant, among which strains resistant to carbapenems are particularly worrisome, as infections caused by such strains may limit the choice of existing antibiotics. We conducted a nationwide and genome-based surveillance on the prevalence and antibiotic susceptibility profile of carbapenem-resistant *A. baumannii* (CRAB) strains collected from intensive care units (ICUs) in hospitals in different provinces and investigated the routes of transmission and mechanism of resistance by whole-genome sequencing and phylogenetic analysis. We found that CRAB strains were prevalent in 71.4% (55/77) of the ICUs surveyed. Clonal spread of CRAB was found in 37.6% (29/77) of ICUs and a total of 22 different clones were identified. Most clones were transmissible within one ICU, but up to six clones could be detected in at least three hospitals. In addition, carbapenem-hydrolysing class D β-lactamases (CHDL) were found to be mainly responsible for carbapenem-resistance in *A. baumannii* and the ST2 global-clone is the predominant type of CRAB in China. Importantly, we found that CRAB isolates currently exhibited an extremely low rate of resistance to colistin (0.4%) and tigecycline (2.5%), but a high rate of resistance to ceftazidime–avibactam (70.2%). Findings in this work shall facilitate development of appropriate antimicrobial regimens for treatment of CRAB infections. Further surveillance and research on the evolutionary and epidemiological features of clinical CRAB strains are necessary.

## Introduction

*Acinetobacter baumannii* is an important Gram-negative pathogen that often causes serious nosocomial infections, especially among immunocompromised and elderly patients in intensive care units (ICUs) [1]. *A. baumannii* can cause various infections in the respiratory tract and bloodstream, as well as wound infections and meningitis. Strains of this bacterial species also asymptomatically colonize the intestinal and respiratory tracts and skin [2]. Besides, *A. baumannii* are known to be resilient to desiccation and remain viable for several days on dry inanimate surfaces [3]. In the past two decades, carbapenem antibiotics such as imipenem and meropenem have become the primary choice for treatment of bacterial infections as a result of widespread dissemination of multidrug-resistant organisms, especially in the hospital environment [4,5]. The rate of resistance of clinical *A. baumannii* strains to carbapenems has also increased significantly. Data from the Antimicrobial Surveillance Network (CHINET) showed that the proportion of *A. baumannii* strains that are resistant to meropenem and imipenem increased from 30.1% and 39.0% (2005) to 71.5% and 72.3% (2021), respectively. Production of oxacillinases (Ambler class D β-lactamases), especially OXA-23, was shown to be the key mechanism of resistance in these carbapenemase-hydrolyzing *A. baumannii* strains [6,7]. A recent systematic review and meta-analysis showed that 60% ∼ 87% of *A. baumannii* strains that cause hospital-acquired (HAP) and ventilator-associated pneumonia (VAP) are multi-drug resistant, and that such infections, which pose challenges to clinical treatment, are associated with high mortality rate, increased treatment cost, long duration of hospitalization, and limited therapeutic choices [8].

Colistin and tigecycline are often considered the most potent first-line agents used to treat infections caused by CRAB and other multidrug-resistant (MDR) strains [9,10]. Although most CRAB strains remain highly sensitive to colistin and tigecycline, there has been an increasing number of reports on colistin and tigecycline-resistant *A. baumannii* in recent years [6–12]. To date, mutations in the *pmrA/pmrB* and *lpxACD* genes are the principal mechanisms of resistance to colistin; on the other hand, mutational changes in the *tet*(X) gene and over-expression of resistance-nodulation-cell division (RND) efflux pumps are the major mechanisms of tigecycline resistance observed in *A. baumannii* isolates [13–15]. Currently, over 50% of clinical *A. baumannii* strains exhibit resistance to ceftazidime–avibactam (CAZ-AVI), which is a novel *β*-lactam-diazabicyclooctenone *β*-lactamase inhibitor combination approved by the FDA for treatment of complicated intra-abdominal and urinary tract infections in US [16,17].

Few epidemiological studies on CRAB strains have been performed in China, especially for those recovered from ICUs. Here, we conducted a genome-based nationwide survey of the prevalence of CRAB in 77 hospital ICUs in China and investigated the molecular epidemiological features, mechanisms of resistance to colistin, tigecycline, and CAZ-AVI, as well as the transmission characteristics of these strains, in order to identify critical factors responsible for causing an increase in prevalence of CRAB infections in ICUs in China. Findings in this work shall provide important insight into development of effective strategies for worldwide control of CRAB and minimize the incidence of untreatable infections in clinical settings.

## Materials and methods

### Bacterial strain collection and species identification

A total of 1,072 faecal swabs and 1,030 sputum specimens were collected from 1,097 patients in the ICUs of hospitals located in 17 provinces (Anhui, Hainan, Fujian, Hebei, Guangdong, Henan, Guangxi, Guizhou, Hubei, Jiangxi, Hunan, Liaoning, Shandong, Sichuan, Shanxi, Yunnan, and Zhejiang) and three municipalities (Chongqing, Shanghai, and Tianjin) in China during the period of June to September in 2020. Among the 1,097 patients, 1,005 provided both faecal swabs and sputum specimens; 67 and 25 patients only supplied faecal swabs and sputum samples, respectively. *A. baumannii* strains were identified by matrix-assisted laser desorption/ionization time of flight mass spectrometry (MALDI-TOF MS) (Bruker, Bremen, and Germany). Antimicrobial susceptibility of the test strains was determined by the broth microdilution method according to Clinical and Laboratory Standards Institute (CLSI) guidelines [18], with the reference strain *E. coli* ATCC 25922 being used as the quality control.

### Quantitative real-time PCR (qRT-PCR) assay

The expression level of *bla*_OXA-23_ in strains R4-1 and R6-1 was evaluated by qPCR. Total RNA of the strains R4-1 and R6-1were extracted and purified by using the Qiagen RNeasy mini kit and Turbo DNA free kit (Invitrogen) according to the manufacturer’s instructions. Reverse transcription was performed by using the SuperScript III quantitative one-step kit (Invitrogen), with *rpoB* being used as a reference gene to normalize the expression levels of test genes. Primers used in qPCR are listed in Table S1. Quantitative real-time PCR assay was performed by using the QuantStudio 7 Real-Time PCR System (Applied Biosystems) and SYBR Green master mix (Applied Biosystems). The tests were performed in triplicates. The results of qRT-PCR were analyzed by the Design & Analysis Software 2.6.0 and visualized by GraphPad prism V8.0.1.

### Whole genome sequencing

Total bacterial DNA of 256 clinical carbapenem-resistant *Acinetobacter* isolates were extracted using Pure-Link genomic DNA mini kit (Invitrogen, USA). DNA library preparation was constructed by using the NEBNext Ultra DNA library prep kit for Illumina (NEB). Whole genome sequencing was conducted on an Illumina Hiseq X sequencing platform. Raw genome sequences were trimmed and quality-filtered using Trimmomatic v0.38 [19]; draft sequences were assembled by SPAdes 3.13.1 [20]. Long-reads DNA preparation was using the Rapid Barcoding Sequencing kit SQK-RBK110.96 (Oxford Nanopore, UK). The optional purification and concentration step was processed by using the Agencourt AMPure XP system (Beckman Coulter, USA) following the manufacturer’s manual. Sequencing was then conducted by using the Nanopore MinION platform and the MinION flow cell (R9.4.1 FLO-MIN106, Oxford Nanopore) [21]. Raw data was collected using the MinKNOW software v22.05.5. All ONT read sets were basecalled and demultiplexed using Guppy v6.1.7 and adapters on the ends of reads were trimmed off and reads with internal adapters were discarded using Porechop 0.2.4. The complete genome sequences of R4-1 and R6-1 were acquired by assembling the short and long reads obtained from both Illumina and Nanopore platforms through Unicycler v0.4.9b [22]. Multilocus Sequence Typing (MLST) and core-genome MLST (cgMLST) of the test isolates based on the multilocus sequence typing databases (http://github.com/tseemann/mlst) and SeqSphere+v3.4.0 (http://www.ridom.de/seqsphere/), respectively, were performed. The assembled genome was annotated using the RAST server [23]. To screen for antimicrobial resistance (AMR) genes and mobile elements, draft genome maps were produced by BLAST (http://blast.ncbi.nlm.nih.gov/Blast), ResFinder [24], ISfinder (https://www-is.biotoul.fr/) and the CLC Genomics Workbench through manual setting (identity 98%, coverage 60%).

### Phylogenetic analysis

Trimmed and quality-filtered assembly sequences acquired from 245 CRAB strains were aligned to *A. baumannii* strain 21 initially collected from Zhejiang province in this study. Single-nucleotide polymorphisms (SNPs) were identified by Snippy v3.1 with default settings [25], which utilized BWA-MEM v0.7.12 for short read alignment. Snippy produced two files known as “core-aln” and a “core-full-aln”. The “core-full-aln” profile was then subjected to ML phylogenetic analysis using Fasttree v2.1.10 [26]. The phylogenetic tree was graphically depicted by iTOL version 3 [27]. MLST and core-genome MLST (cgMLST) type analysis were also performed through clustering analysis of a known MLST-type strain and 14 cgMLST-types among the 245 CRAB isolates [28].

### Ethics approval statement

All studies were approved by the Human Research Ethics Committee (HREC) of the Second Affiliated Hospital of Zhejiang University School of Medicine in May 2020, with the reference no. I2020001370.

## Results

### Epidemiological study of carbapenem-resistant *Acinetobacter* strains in China

**Figure 1.**
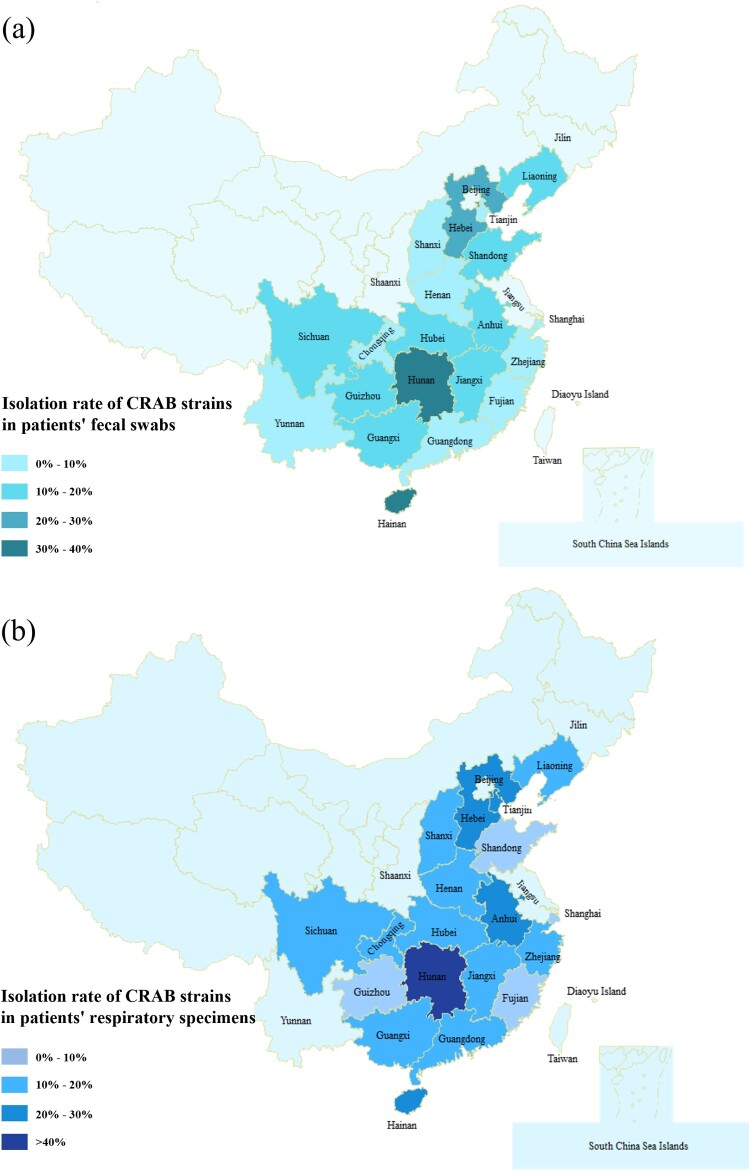
Prevalence of CRAB strains in different provinces of China. (a) The prevalence of CRAB strains recovered from the faecal swabs of patients in different provinces of China. (b) The prevalence of CRAB strains recovered from the respiratory samples of patients in different provinces of China. Different shades of colour represent different prevalence levels of carbapenem resistance.

A total of 256 non-duplicate carbapenem-resistant *Acinetobacter* strains were recovered from 2,102 clinical samples collected from 77 hospital ICUs in 20 provinces and municipalities in China during the period of July to September in 2020. The 2,102 clinical specimens included 1,030 sputum samples and 1,072 faecal swab samples. Results showed that the detection rate of carbapenem resistant *Acinetobacter* strains in the sputum specimens was higher than that of faecal swabs. Among the 1,030 sputum specimens tested, 156 were found to contain carbapenem resistant *Acinetobacter* strains (15.1%); for faecal specimens, the positive rate was 9.3%, with 100 of the 1,072 specimens being found to contain carbapenem resistant *Acinetobacter*. Among the carbapenem-resistant *Acinetobacter* strains, 245 isolates were identified to be CRAB and the remaining 11 isolates were *A. pitti*. According to our data, carbapenem resistant *Acinetobacter* strains were highly prevalent (77%, 55/77) in ICUs across China, but significant variation in isolation rate was observed among different provinces, with the lowest in Fujian (2.9%) and the highest in Hunan province (70%) (Figure 1). The 245 CRAB strains were subjected to further analysis.

### Susceptibility of CRAB strains to various antimicrobial agents

**Figure 2.**
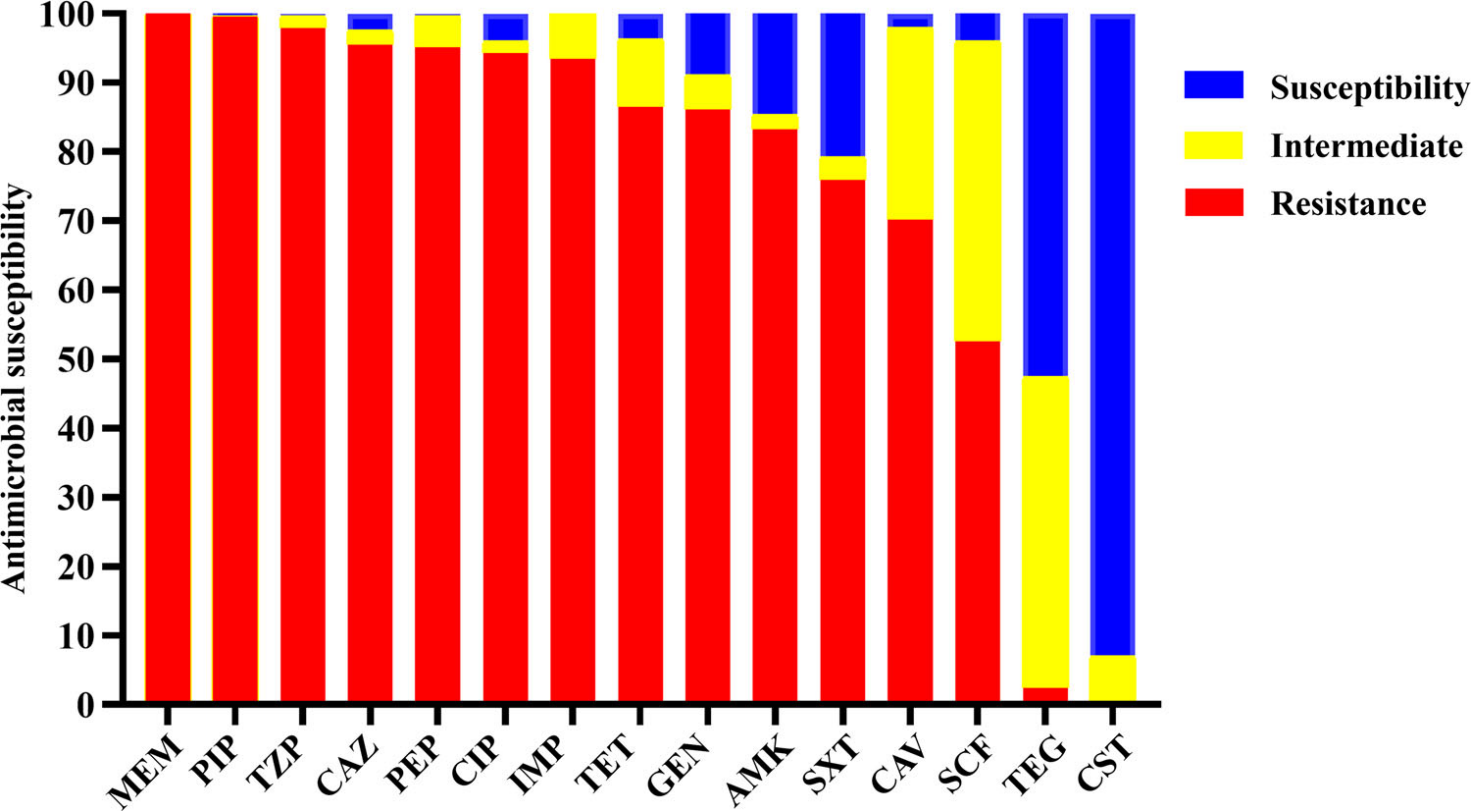
Antimicrobial susceptibility of 245 CRAB strains isolated from ICUs. IMP, imipenem; MEM, meropenem; CAZ, ceftazidime; SXT, trimethoprim/sulfamethoxazole; TZP, piperacillin/tazobactam; CAV, ceftazidime–avibactam; PIP, piperacillin; FEP, Cefepime; CHL, chloramphenicol; CST, colistin: TGC, tigecycline; CIP, ciprofloxacin; GEN, gentamicin: AMK, amikacin; TET, tetracycline; SCF, cefoperazone–sulbactam. Interpretations of resistance phenotypes follow those of the CLSI (Clinical and Laboratory Standards Institute) M100-S26 document.

The majority of the 245 CRAB isolates tested were found to be multidrug resistant, with 94.3% and 86.1% of the strains being resistant to ciprofloxacin and tetracycline, respectively. The rate of resistance to the six *β*-lactam drugs tested, namely meropenem, piperacillin, piperacillin/tazobactam, ceftazidime, cefepime, and imipenem, was 100%, 99.6%, 97.9%, 95.5%, 95.1%, and 93.5%, respectively. Intermediate resistance was observed among the others. The rate of susceptibility to ceftazidime–avibactam, gentamicin, amikacin, sulfamethoxazole/trimethoprim, and cefoperazone–sulbactam were 29.8%, 13.9%, 16.7%, 24.1%, and 49.7%, respectively. Resistance to tigecycline and colistin was extremely rare among CRAB isolates in China, accounting for only 2.45% and 0.4% of the test strains, respectively (Table 1, Figure 2).

**Table 1.** Genetic characteristics and antimicrobial susceptibility of CRAB strains tested in this study.

Categories of Antibiotics	Antimicrobial agent	Breakpoints susceptibility /resistance	MIC_50_	MIC_90_	R(%)	Genetic resistance determinant
	Imipenem	≤2 mg/L / ≥8 mg/L	16	32	93.46%	*bla*_OXA-23_, *bla*_OXA-66_
	Meropenem	≤2 mg/L / ≥8 mg/L	32	64	100.00%	
β-lactamases-antibiotics	Ceftazidime	≤8 mg/L / ≥32 mg/L	64	128	95.51%	*bla*_OXA-23_, *bla*_OXA-66_
	Cefepime	≤8 mg/L / ≥32 mg/L	64	128	95.10%	
	Piperacillin	≤16 mg/L / ≥128 mg/L	>128	>128	99.59%	*bla*_TEM-1_
	Piperacillin/tazobactam	≤16/4 mg/L / ≥128/4 mg/L	>256/4	>256/4	97.95%	IS*Aba1*-*bla*_OXA-23_ or *bla*_OXA-66_
	Ceftazidime–avibactam^a^	\	32/4	>32/4	70.2%	Over expression of *bla*_OXA-23_
	Cefoperazone–sulbactam^b^	\	64/32	128/64	50.26%	Over expression of *bla*_ADC-25_
Polymyxins	Colistin	≤2 mg/L / ≥4 mg/L	1	1	0.41%	*pmrC*^S354A^
Quinolone	Ciprofloxacin	≤1 mg/L / ≥4 mg/L	>16	>16	94.28%	*gyrA*^S81L^; *gyrB*^A414T^; *parC*^S84L^
Aminoglycosides	Gentamicin	≤4 mg/L / ≥16 mg/L	>64	>64	86.12%	*aph(3’), aac(3’), armA*
	Amikacin	≤16 mg/L / ≥64 mg/L	>128	>128	83.26%	
Tetracycline	Tigecycline^c^	\	1	2	2.49%	mutation in RND family efflux pump genes
	Tetracycline	≤4 mg/L / ≥16 mg/L	>64	>64	86.12%	*tet(39)*, *tet(B)*(Mainly)
Folate inhibitors	Trimethoprim/sulfamethoxazole	≤2/38 mg/L / ≥ 4/76 mg/L	>64/1216	>64/1216	75.91%	*sul1, sul2*

^a^ No susceptibility breakpoint for *Acinetobacter* has been provided by CLSI. Susceptibility and resistance of Enterobacteriaceae to Ceftazidime and avibactam combination, which are ≤8/4 mg/L and ≥32/4 mg/L, respectively, are used as reference values.

^b^ No susceptibility breakpoint for *Acinetobacter* has been provided by CLSI. CLSI breakpoints for Enterobacteriaceae susceptibility and resistance to Cefoperazone–sulbactam, which are ≤16/4 mg/L and ≥64/4 mg/L, respectively, are used as reference.

^c^ No susceptibility breakpoint for *Acinetobacter* has been provided by CLSI. The FDA-approved breakpoints for Enterobacteriaceae susceptibility and resistance to tigecycline are ≤2 mg/L and ≥8 mg/L, respectively.

### Phylogenetic analysis of clinical CRAB isolates in China

**Figure 3.**
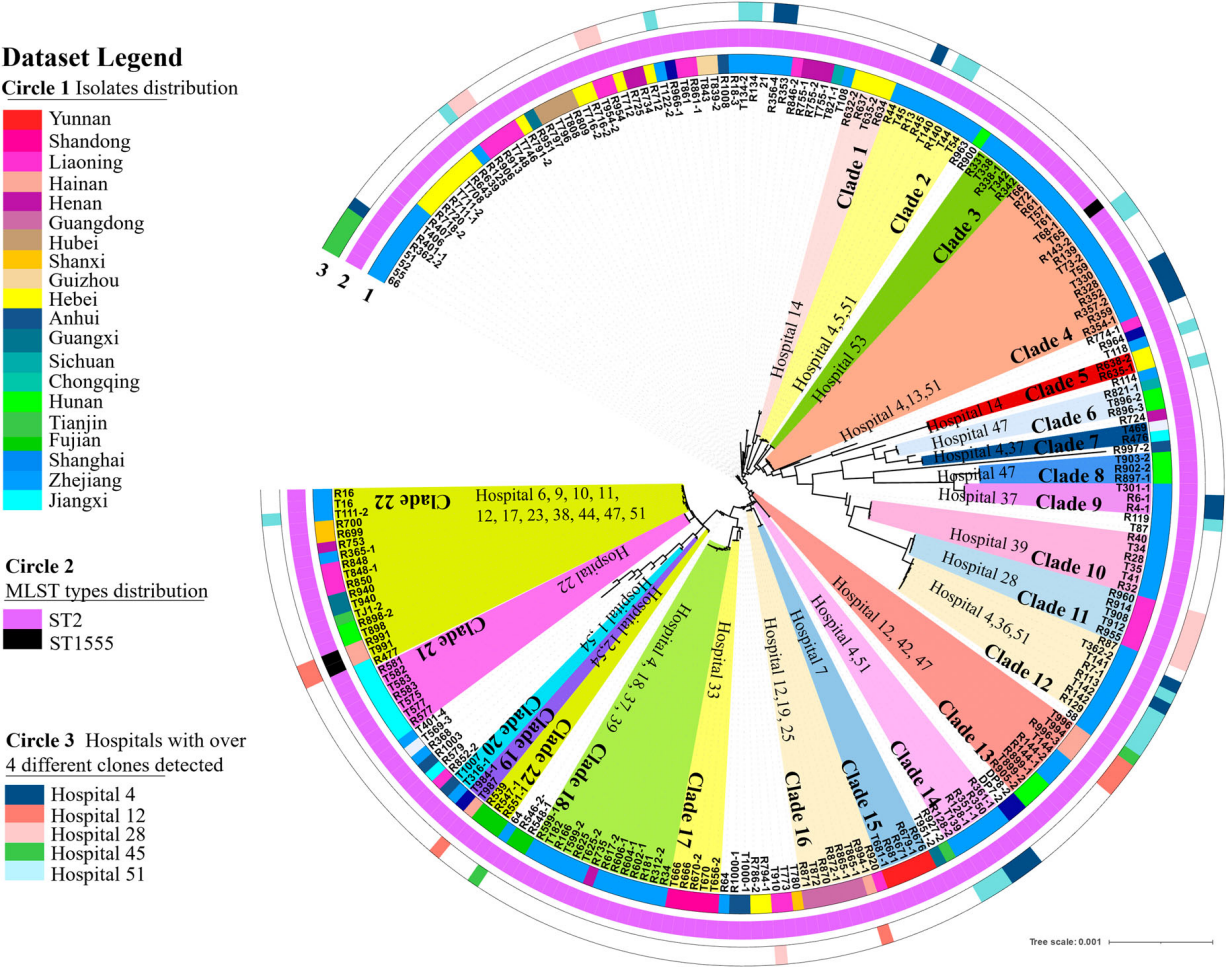
Phylogenetic tree of 245 CRAB strains collected from ICUs in various parts of China during the period July to September 2020. Circle 1 depicts strains isolated from various provinces or municipal cities in China; Circle 2 denotes distribution of MLST types. Strains in each clade are depicted in the same colour and are regarded as clonally disseminated. Circle 3 depicts 5 ICUs in which four or more clones were recovered.

**Figure 4.**
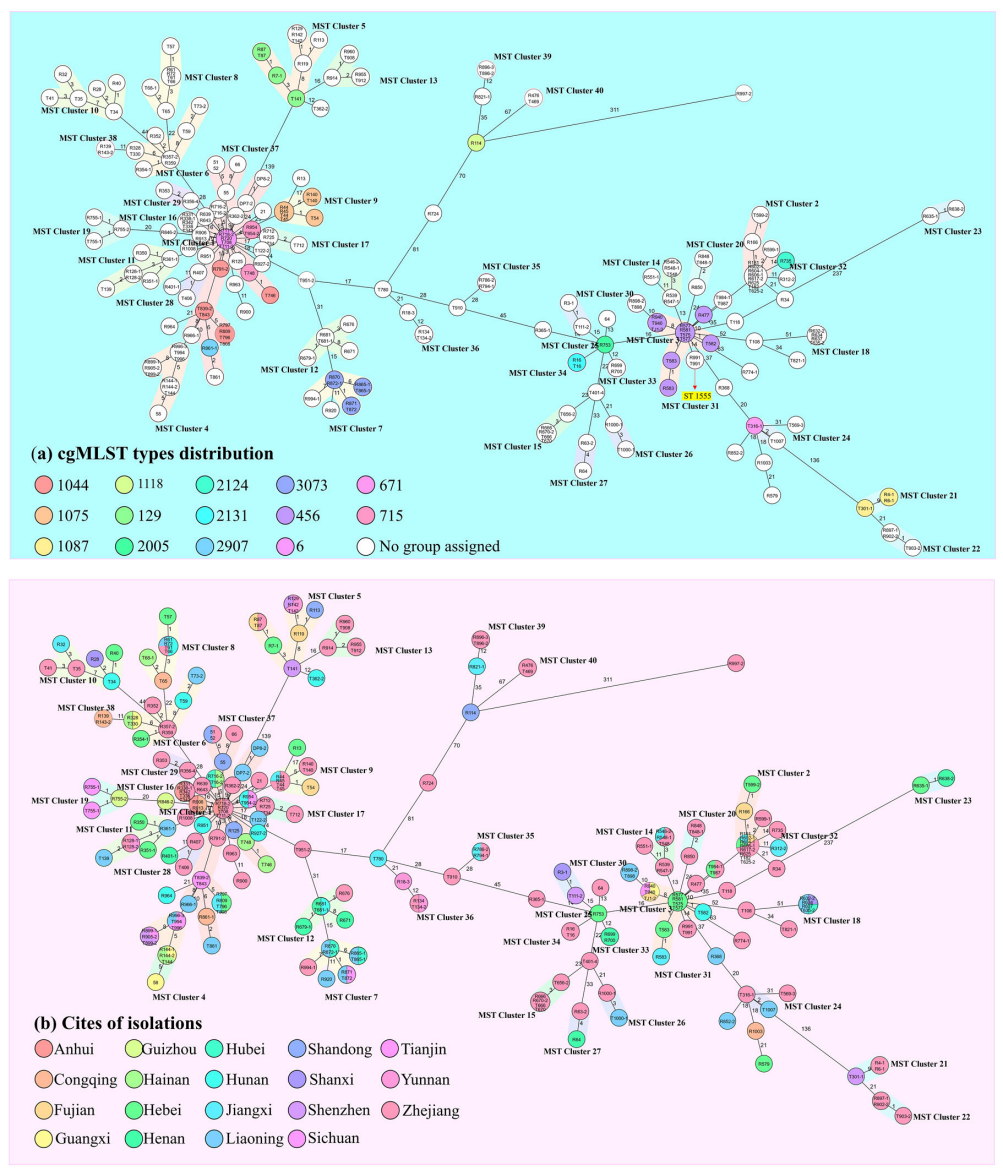
Minimum spanning tree constructed on the basis of cgMLST allelic genes of 245 CRAB strains isolated from ICUs in China. Each circle depicts an allelic profile based on sequence analysis of 2050 cgMLST genes. The length of the connecting lines represents the number of target genes with different alleles. (a) Colours of the circles denote different cgMLST types. Closely related genotypes (less than 10 alleles difference) are shaded in same node, and clusters are numbered consecutively (1–40). Red arrows indicate that strains belonged to ST1555, the remaining strains are all identified as ST2 type (b) Colours of the circles represent different isolation sources.

To further investigate the transmission and distribution features of clinical CRAB strains in ICUs in China, phylogenetic tree was created for the 245 CRAB isolates (Figure 3). Pairwise SNP distance is an important factor that infers putative clonal transmission of bacteria; clonal spreading inference parameters (for meticillin-resistant *S aureus*, ≤25 SNPs for other species including *E coli, Enterococcus faecium, K pneumoniae strains*) were defined in a recent study [29]. There is no clear range for transmission inference threshold of the pairwise SNPs distances in *A. baumannii* strains. However, 14 pairs of such strains collected from a same patient were found to exhibit a range of 1 ∼ 40 SNPs differences from each other [29], suggesting that *A. baumannii* strains have a higher transmission inference threshold. SNPs distances of pair strains less than 100 might be genetically related clones or closely related evolutionary strains. Clustering analysis showed that 62.5% of the strains belonged to a total of 22 clonal dissemination clades which exhibit a difference of less than 100 SNPs (Tables S3–S24). These 22 clonal dissemination clades were collected from 29 of a total of 77 ICUs. Clonal transmission and evolution were observed in 17 out of the 77 (22.0%) ICUs. CRAB strains in clades 22 exhibited more extensive dissemination across different hospitals when compared to strains in other clades. These strains were detected in 12 ICUs in hospitals of 10 provinces (Table S24). Clones with high potential of dissemination should be of concern and deserve further study. The strains from each ICU were normally genetically related, with less than 2 genetically distinct clones identified. In five hospitals, however, more diverse types of strains were detected. In addition, the traditional Pasteur scheme of multilocus sequence typing (MLST) analysis showed that ST2 (*cpn60*-2*, fusA*-2*, gltA*-2*, pyrG*-2*, recA*-2*, rplB*-2, and *rpoB*-2) was predominant (243/245, 99.2%). The reminding 2 strains were found to belong to ST1555 (*cpn60*-2*, fusA*-2*, gltA*-181*, pyrG*-2*, recA*-2*, rplB*-2, and *rpoB*-2) which exhibited close genetic relationship with ST2 strains (Figure 3). On the other hand, high resolution cgMLST analysis showed that these 245 CRAB strains could be grouped into 40 distinct clades, which included 14 known cgMLST types and 26 unassigned groups with genetic difference of less than 10 alleles (Figure 4(a)).

These 40 sub-clades were closely related to the two cgMLST types (cgST6, cgST456). To date, the *bla*_OXA-23_ gene has been detected in five transposons including Tn2006, Tn2007, Tn2008, Tn2008B, and Tn2009 [30]. cgMLST analysis showed that the transposon of *bla*_OXA-23_ displayed a strong structural relationship with these two taxa. Strains that are genetically close to cgST456 normally contain a Tn2006 within the structure of *_Δ_*IS*Aba1-bla*_OXA-23_-ATPase-*res-_Δ_*IS*Aba1*. The cgST6-linked isolates harboured a Tn2009 element and comprised the structure of *_Δ_*IS*Aba1-bla*_OXA-23_-ATPase-*hp-parA-yeeC-hp-yeeB-_Δ_*IS*Aba1*. Besides, strains closely related to cgST456 in MST tree have the potential to acquire a mega plasmid such as pTG22653 (GenBank accession number CP039519.1), of which the detection rate was 28% (21/75). Further studies are warranted to investigate the underlying genetic transfer mechanisms concerned. On the other hand, the known cgMLST-type strains can be observed in the trunk of the minimum spanning tree (MST) and the non-assigned groups are in the branches. Meanwhile, the MST containing isolation source information could better depict the geographical distribution and relationship between these ST2 bacteria (Figure 4(b)). In particular, strains collected from Zhejiang province could be observed in almost all branches and were found to exhibit substantial genetic diversity. Up to 468 different alleles were detectable among these isolates, and were all assigned to the ST2 type.

Importantly, cgMLST analysis can be more intuitively applied to study the clonal spread of clinical CRAB strains. A total of 25 sets of MST nodes of strains collected from different provinces shared identical allelic profiles (Figure 4(b), different colours in one circle), suggesting that clonal dissemination not only occurred in same ICU, but the strains involved were also detectable in various hospitals in different provinces. In fact, as much as 88% (22/25) of strains bearing these nodes were collected from two separate provinces; for the remaining clonally spreading strains, those belonging to two nodes were collected from three provinces, and one strain could be found in four provinces. Meanwhile, approximately half of the strains that exhibited clonal distribution were collected from Zhejiang, and the rest were mainly collected from the Jiangxi and Liaoning Province. However, most of these colonies could not be assigned to any groups in cgMLST analysis and were found disseminated in various branches of the MST, indicating that CRAB strains in China which were closely associated with the known cgMLST types continue to evolve. The microevolutionary events of CRAB strains can create sub-clones, such trend of evolution might pose further challenges to treatment of clinical infections.

### Characterization of resistance gene profiles in CRAB isolates

**Figure 5.**
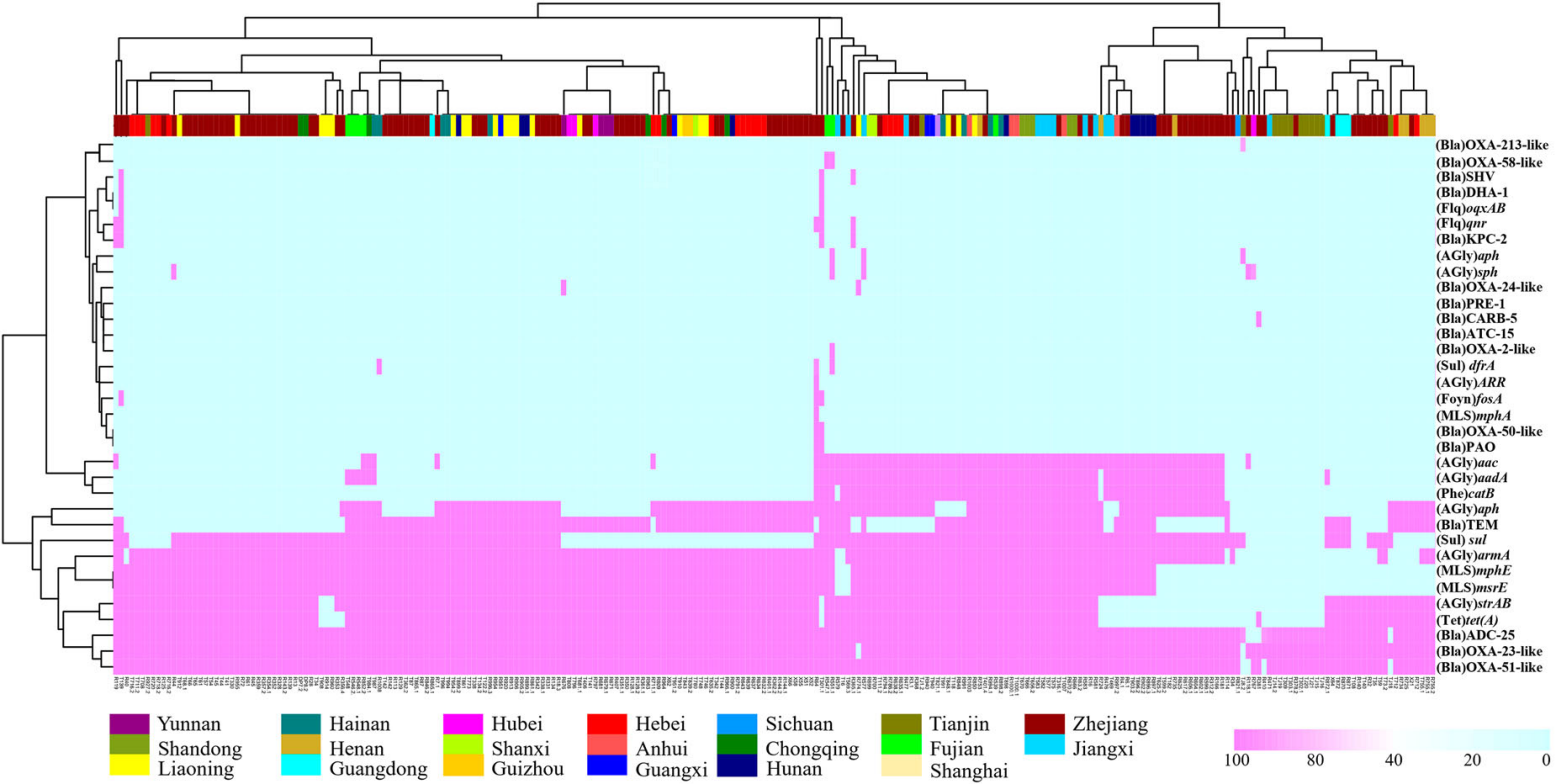
AMRs gene analysis of 245 CRAB strains collected nationwide in China. Heatmaps were obtained by aligning the draft genome sequence of each strain to the AMR gene database. *Acinetobacter baumannii* are clustered using a maximum likelihood tree. Red and light cyan blue depict the presence and absence of AMRs in the test strains, respectively. The right side denotes the category of AMRs. The colour bar above depicts the geographical distribution of CRAB isolates, with each region being shown by a specific colour in the dataset legend.

A total of 34 antimicrobial resistance genes (ARGs) were detectable in the 245 CRAB isolates, conferring resistance to eight categories of antibiotics (Figure 5); 10 of the ARGs were harboured by over 50% of strains, including *aph* (56.8%), *armA* (81.6%), *bla*_ADC-25_ (81.6%), OXA-23-like (94.2%), OXA-51-like (93.8%), *mphE* (77.8%), *msrE* (77.8%), *strAB* (77.8%), *sul* (67.9%), and *tet* (78.2%). These genes confer resistance to five classes of antibiotics: *β*-lactams, aminoglycosides, macrolides, sulphonamides, and tetracyclines. Detail information were listed in (Table 1). In addition, four functional groups of OXA *β*-lactamases encoded by 10 variants of the *bla*_OXA_ gene, which are known to be commonly harboured by CRAB strains, were identified (Table S2). The distribution of AMRs exhibited no obvious geographical specificity. The *mcr-1* and *tet(X)* genes, which conferred resistance to the last-resort antibiotic colistin and tigecycline, respectively, were not found in any of the CRAB strains tested in this nationwide survey.

### Characterization of carbapenemase-encoding genes in clinical CRAB isolates

**Figure 6.**
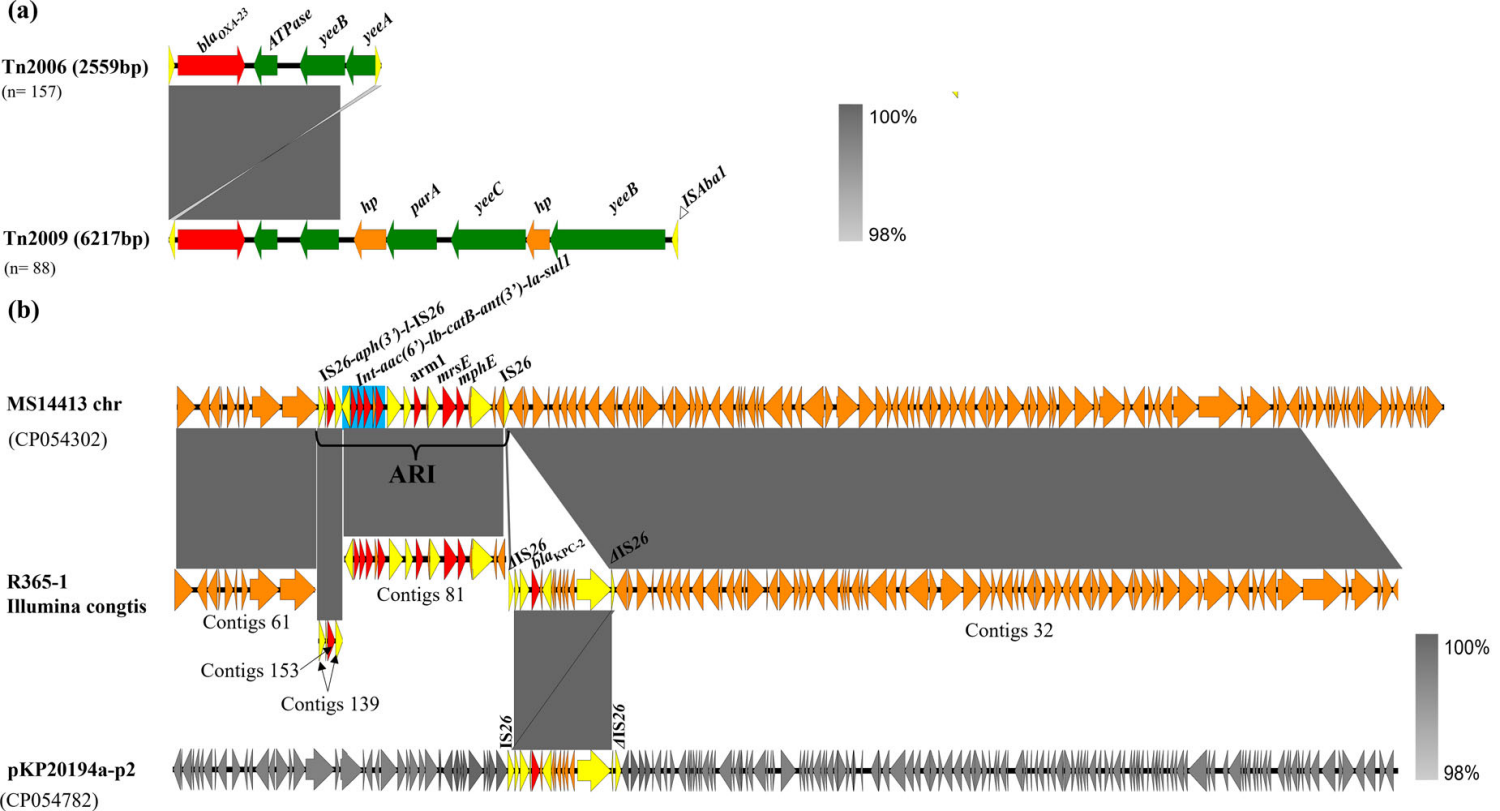
Genetic environment of acquired *bla*_OXA–23_ and *bla*_KPC-2_ elements in CRAB isolates created by Easyfigure. (a) Structure alignment of Tn2006 and Tn2009. Red depicts *bla*_OXA-23_ genes; Green and yellow denote putative functional protein and insertion sequence, respectively. (b) Linear alignment of chromosomal fragments MS114413-chr, R365-1 matching Illumina contigs and plasmid pKP20194a-p2. Mobile elements are highlighted in yellow and drug-resistance gene are depicted by red arrows. Blue frame depicts class I integron. Plasmid backbone of pKP20194a-p2 is highlighted in grey.

The 245 CRAB isolates were further tested for their capacity to produce carbapenemase and carriage of carbapenemase-encoding genes. WGS analysis showed that all carbapenem-resistant isolates that belonged to *A. baumannii* produced carbapenemases. A variety of carbapenemase encoding genes were found in these CRAB strains, among which the acquired OXA-23 and intrinsic OXA-51 enzyme were key determinants of carbapenem resistance, with a detection rate of *bla*_OXA-23_ approaching 98.7% (242/245). BLASTN indicated that the *bla*_OXA-23_ gene and relevant variants resided exclusively in the Tn2006 and Tn2009 elements, the size of which were ∼2599 bp (*n* = 157) and ∼6217 bp (*n* = 88), respectively (Figure 6(a)). Tn2006 comprised four predicted coding sequences (CDs) with a CG content of 36.9% and a genetic environment of *_Δ_*IS*Aba1-bla*_OXA-23_-*ATPase*-*yeeB*-*yeeA-_Δ_*IS*Aba1*. The truncated IS*Aba1* element located at each side was 83 bp in size and exhibited a high degree of sequence homology with each other. However, the structure of Tn2009 was *_Δ_*IS*Aba1-bla*_OXA-23_-ATPase-*hp-parA-yeeC-hp-yeeB-_Δ_*IS*Aba1*, which was flanked by two truncated IS*Aba1* elements of 77 bp in size. Such Tn2009 element has only been reported in China [30].

In addition, four CRAB isolates (1.5%) were shown to carry the *bla*_KPC-2_ determinant (Figure 5). *Klebsiella pneumoniae* carbapenemase (KPC) production *by A. baumannii* has rarely been reported in China since it was first discovered in Puerto Rico [31]. Sequence alignment indicated that the *bla*_KPC-2_ gene was located in a contig with the size of ∼96 kb, among which a ∼10 kb exogenous fragment with the core structure of IS*Kpn27*-*bla*_KPC-2_-IS*Kpn6* bordered by IS*26* at both ends exhibited a high degree of sequence homology (100% identity, 100% coverage) with the corresponding segment in plasmid pKP20194a-p2 (Accession no. CP054782) and was thus considered originated from *Klebsiella pneumoniae*. BLAST analysis also showed that the remaining ∼86 kb of this contig was homologous (100% identity, 100% coverage) to its counterpart in the chromosome of *A. baumannii* MS14413 (Accession no. CP054302), suggesting the *bla*_KPC-2_ gene could be integrated into the ARI region of the chromosome of *A. baumannii* isolates by genetic recombination mediated by mobile genetic elements such as IS*26* (Figure 6(b)).

### Mechanisms of colistin, tigecycline and CAZ-AVI resistance in CRAB strains

Colistin and tigecycline are the potential choices of antibiotics for treatment of CRAB strains infections [32]. CRAB strains resistant to these two antimicrobial agents are sporadically observed in this study. One strain, namely R44, was resistant to colistin. However, the *mcr-1* gene was not detectable in this strain and its resistance phenotype could not be transferred in filter mating assay, hence we speculated that the resistance phenotype of this strain was due to mutations in lipo-oligosaccharide (LOS)-modifying genes. BLAST analysis and comparison with the colistin-susceptible (ColS) isogenic CRAB strains R45, T44, and T45 subsequently confirmed that this strain harboured a *pmrC*^S354A^ mutation. We hypothesize that this mutation is the underlying cause of colistin resistance in this strain. In addition, six isolates were found to exhibit tigecycline resistance due to expression of RND-type efflux pumps encoded by the *adeABC*, *adeFGH*, and *adeIJK* genes. Among them, four types of mutations in *adeS* and *adeR* genes, which encode the Ade two-component signalling system, were detectable in four strains. These include the *adeS*^K131N&L29R^ double mutations, truncation by IS*Aba1* in the *adeS* gene and the *adeR*^D208N^ mutation. The *adeR* and *adeS* genes are known to play a role in regulating the expression of AdeABC efflux pumps [33]. On the other hand, the *adeN* gene is involved in regulating expression of AdeIJK efflux pumps. Truncation of the *adeN* gene by IS*Aba1* might therefore abolish the regulatory function and results in expression of tigecycline resistance in strain R64. Another mutation in the *adeH* gene (*adeH*^Q415H^), whose product constitutes the AdeFGH efflux pump, could be detected in T142-bearing variants in which the *adeS* gene was truncated by a IS*Aba1* element.

Moreover, CRAB isolates exhibited high (70.2%) and intermediate (27.7%) level of resistance to CAZ-AVI; such phenotype strongly correlated with carbapenem resistance, in which over-expression of the *bla*_OXA–23_ gene was considered a key mechanism of CAZ-AVI resistance in CRAB strains. This chromosomal genetic element is inherent to *A*. *baumannii* and readily over-expressed as a result of promoter activation by insertion of the IS*Aba1* element. In addition, multiplication of *bla*_OXA-23_ in *A. baumannii* is also a mechanism of resistance to CAZ-AVI. A pair of isogenic strains, namely R4-1 (CAZ-AVI-susceptible) and R6-1(CAZ-AVI-resistant), which belonged to the cgST1087 type with less than 10 SNPs difference, were subjected to assessment of expression level of *bla*_OXA–23_ to investigate the degree of contribution of carbapenemase genes to CAZ-AVI resistance. Higher expression level of *bla*_OXA–23_ was observed in R6-1 when compared to R4-1 according to results of RT–PCR (Figure S1). Furthermore, a 7 kb circular intermediate with a structure of *bla*_OXA-23_-ATPase-*hp-parA-yeeC-hp-yeeB-*IS*Aba1*, as well as a ∼1 kb circular intermediate harbouring a copy of IS*Apl1*, were identified in strain R6-1 by hybrid assembly with Unicycler. Nanopore raw reads analysis showed that the *bla*_OXA-23_ gene was located in a 7kb-repeats region in each of two copies of a chromosomal fragment (Figure S2). In contrast, strain R4-1 with a single copy of Tn2009 in the chromosome was not found to contain such circular intermediate in sequence analysis. Other mechanisms of CAZ-AVI resistance were also closely associated with production of OXA carbapenemase, as the *bla*_OXA-72_ gene was detected in the remaining *bla*_OXA-23_ negative strains.

## Discussion

*Acinetobacter baumannii* is an important Gram-negative pathogen that often causes infections with high mortality, especially in nosocomial settings. Due to incessant use of carbapenems including meropenem and imipenem in treatment of bacterial infection, CRAB strains have emerged and are recently considered as one of the most problematic bacterial pathogens. CRAB have become highly prevalent worldwide, including Asia-Pacific, the Indian continent, North America, Latin America, and Europe [34]. Worryingly, reports of CRAB strains are often associated with outbreaks in hospital ICUs and pose severe challenge to infection control in healthcare facilities [35,36]. Over 8,500 infections cases and 700 deaths due to CRAB strains were reported in United State in 2017 [37]. CRAB surveillance in hospitals has been implemented in China for many years. Data from the CHINET surveillance network depicted the increasing trend of CRAB strains among clinical *A. baumannii* isolates in past two decades. Based on the available data, a heatmap showing the distribution of CRAB in various provinces around China has been generated (https://www.chinets.com/Data/GermYear).

Findings of this work confirmed the epidemiological features of CRAB strains recovered in ICUs across China. Importantly, 71.4% (55/77) ICUs were found to be contaminated by CRAB strains, with detection rates of 9.5% and 15.2% in faecal and sputum samples, respectively. Only 4.4% (44/1005) patients in ICUs had CRAB strains in both specimens. The high isolation rate of CRAB in these two types of specimen represent a significant threat to patients as bacteria colonized in upper respiratory tract and gastrointestinal tract may cause ventilator-associated pneumonia (VAP) [8,38]. Significant variation in isolation rate was observed in different provinces, with Fujian province (2.9%) being the lowest and Hunan (70%) being the highest. Almost all strains were found to be the ST2 type, which is a common type reported in China and also the most prevalent clonal type reported globally and documented in various databases [39]. MLST is a general method based on detecting the presence seven pairs of housekeeping genes and commonly used for assessing clonal relationships among bacterial strain since 1998 [40]. However, it might not be applicable for CRAB strains since ST2 is the most prevalent in China [6]. Phylogenetic tree and cgMLST analysis based on SNPs and maximum of core genes exhibited higher resolution than MLST analysis, and have higher application value in analyzing CRAB transmission routes and efficiency in clinical settings [41]. Clustering analysis showed that clinical CRAB strains collected in most ICUs exhibited highly similar genetic features, as 40.3% (31/77) of ICUs had only one clonal strain and 22% (17/77) of ICU had two clonal strains. CRAB strains collected in two ICUs (2/77) exhibited more complex genomic composition, as such strains could be assigned to 13 and 8 different clades of phylogenetic tree, respectively. CRAB strains are normally regarded as a nosocomial pathogen [42]. In this work, we not only observed dissemination of clonal strains (characterized by recovery of isogenic CRAB strains from two more patients) in 22.0% (17/77) of ICUs tested, but also obtained evidence of transmission of clonal strains between hospitals in different provinces, indicating that infection control measures need to be improved in China to reduce the risk of bacterial dissemination in the hospital environment.

Importantly, all the CRAB strains tested in this work exhibited resistance to meropenem, and as much as 93.4% of the strains were also resistant to imipenem; the other 6.6% were found to exhibit intermediate resistance to imipenem (MICs = 4). This is consistent with the observation that the resistance rate of meropenem was slightly higher than that of imipenem over the years in the CHINET surveillance network. This work also confirms that production of the OXA type carbapenem-hydrolyzing-class D (CHDL) *β*-lactamase is the key mechanism for carbapenem resistance in *A. baumannii* in China. Other carbapenemase resistance genes such as *bla*_KPC-2_ was occasionally observed, but the *bla*_NDM_ gene was not detected in these CRAB strains. To date, five transposons containing the *bla*_OXA-23_ gene, including Tn2006, Tn2007, Tn2008, Tn2008B, and Tn2009, have been reported [30]. Tn2006 and Tn2009 carrying *bla*_OXA-23_ and relevant variants were mainly responsible for causing an increase in prevalence of CRAB in China. In contrast, the proportion of colistin resistant CRAB strains has remained low in the past few years [6], whereas *tet(X)*-positive *Acinetobacter* isolates are mostly sensitive to tigecycline [43], suggesting that the sporadic incidence of colistin and tigecycline resistance in China may be mainly caused by mutations, insertional inactivation of LOS-modifying relating gene and over-expression of RND-efflux pump encoding genes, rather than dissemination of MDR plasmids which harboured antimicrobial resistance genes (AMRs) such as *mcr-1* and *tet*(X) genes. On the other hand, the rate of resistance to CAZ-AVI was high among the CRAB strains, presumably as a result of over-expression of *bla*_OXA-23_ via IS*Aba1*-mediated and multiplication mechanisms. Consistently, over 38% of the isolates were found to contain two or more copies of *bla*_OXA-23_ in recent study [44].

In summary, this study conducted a genome-based nationwide surveillance of 245 CRAB isolates collected from various ICUs in China and revealed their epidemiological and molecular features. Key findings of this study are as follows: (1) Nearly 70% (55/77) of ICUs are contaminated by CRAB strains and more than half of the test strains exhibited genetic relationship with each other; a total 22 genetic clones were identified in phylogenetic and SNPs analysis, among which clade 22 has disseminated extensively and deserves further investigation; (2) ST2 is the predominant type among CRAB isolates in China, whereas the remaining strains belonged to ST1555 that also exhibited strong genetic relatedness with ST2 strains; (3) OXA carbapenemase-encoding genes such as *bla*_OXA-23_ and *bla*_OXA-66_ are major determinants of carbapenem resistance in CRAB isolates; and (4) CRAB isolates exhibit a low prevalence of resistance to colistin and tigecycline, and resistance to these two antibiotics is primarily mediated by mutations or insertional inactivation of chromosomal genes; such resistance mechanisms are not readily transmissible. All in all, preventing dissemination and outbreak of CRAB strains in ICUs is a worldwide challenge since such strains pose severe threat to public health. It is necessary to develop inhibitors targeting strains that carry *bla*_OXA-23_–like genes, and undergo surveillance on the evolutionary and epidemiological features of clinical CRAB strains.

## Authors’ contributions

C.C.L and K.C.C contributed equally to this work. C.C.L and K.C.C performed the experiments; H.L., Y.F., J.Y.L., Y.Z. helped with strain and clinical data collection. M.M.X and EWCC helped draft the manuscript. R.Z. and S.C. helped with experiments design and clinical data collection, and supervised the project.

## Supplementary Material

Supplemental Material

## Data Availability

The whole genome sequencing data in this study have been deposited in GenBank under BioProject ID PRJNA734772.
