# Supplementary material for: Epidemiological and genetic characteristics of clinical carbapenem-resistant Acinetobacter baumannii strains collected countrywide from hospital intensive care units (ICUs) in China
Source: Emerg Microbes Infect. 2022 Jul 4;11(1):1730–41. doi: 10.1080/22221751.2022.2093134 (PMC9258068; doi:10.1080/22221751.2022.2093134)
Supplement: Supplemental Material [file TEMI_A_2093134_SM9498.docx]

Supplementary Materials

**Epidemiological and Genetic Characteristics of** **Clinical Carbapenem-Resistant *Acinetobacter*** ***baumannii* Strains Collected Countrywide from Hospital Intensive Care Units (ICUs) in China**

Congcong Liu^1, #^, Kaichao Chen^2,#^, Yuchen Wu^1^, Ling Huang^1,3^, Yinfei Fang^1,4^, Jiayue Lu^1^, Yu Zeng^1^, Miaomiao Xie^2^, Edward Wai Chi Chan^5^, Sheng Chen^2*^, Rong Zhang^1*^

^1^Department of Clinical Laboratory, Second Affiliated Hospital of Zhejiang University, School of Medicine, Zhejiang, Hangzhou, China

^2^Department of Infectious Diseases and Public Health, Jockey Club College of Veterinary Medicine and Life Sciences, City University of Hong Kong, Kowloon, Hong Kong

^3^Department of Clinical Laboratory Medicine, The women's and children's hospital of Linping District, Hangzhou, China

^4^Department of Clinical Laboratory, Jinhua Municipal Central Hospital, Jinhua, China

^5^State Key Lab of Chemical Biology and Drug Discovery, Department of Applied Biology and Chemical Technology, The Hong Kong Polytechnic University, Hung Hom, China

# These authors contributed equally to this work.

Corresponding author:

Sheng Chen, City University of Hong Kong, Kowloon, Hong Kong;

Email: [shechen@cityu.edu.hk](mailto:shechen@cityu.edu.hk).;

Rong Zhang, Department of Clinical Laboratory, Second Affiliated Hospital of Zhejiang University, School of Medicine, Zhejiang, Hangzhou, China;

Email: [zhang-rong@zju.edu.cn](mailto:zhang-rong@zju.edu.cn)

**Supplementary Table S1. qPCR Primers used in this study**

| **qPCR target** | **Primer** | **Sequence (5′–3′)** |
| --- | --- | --- |
| *Bla*_OXA-23_ | OXA-23-RT-F | TATTCTGTATTTGCGCGG |
|  | OXA-23-RT-R | CTATGTGGTTGCTTCTCT |
| *rpoB* | rpoB-RT-F | TCCGCACGTAAAGTAGGAAC |
|  | rpoB-RT-R | ATGCCGCCTGAAAAAGTAAC |

**Supplementary Table 2. Functional groups of the OXA *β*-lactamase-encoding genes and relevant variants in CRAB strains tested in this study.**

| **Acquired OXA sub-family** | **AMRs type** | **n** | **Intrinsic OXA sub-family** | **AMRs type** | **n** |
| --- | --- | --- | --- | --- | --- |
| OXA-23-like | *bla*_OXA-23_ | 242 | OXA-51-like | *bla*_OXA-66_ | 229 |
|  | *bla*_OXA-171_ | 1 |  | *bla*_OXA-67_ | 4 |
|  | *bla*_OXA-225_ | 1 |  | *bla*_OXA-69_ | 4 |
| OXA-24-like | *bla*_OXA-72_ | 1 |  | *bla*_OXA-80_ | 2 |
|  |  |  |  | *bla*_OXA-88_ | 1 |
|  |  |  |  | *bla*_OXA-91_ | 1 |
|  |  |  |  | *bla*_OXA-120_ | 2 |

**Supplementary Table 3. SNP analysis of CRAB isolates in clade1**

| **Hospital** | **Strains** | **R632-2** | **R634** | **R637** | **T635-2** |
| --- | --- | --- | --- | --- | --- |
| **Hospital14** | **R632-2** | 0 | 5 | 5 | 5 |
| **Hospital14** | **R634** | 5 | 0 | 0 | 0 |
| **Hospital14** | **R637** | 5 | 0 | 0 | 1 |
| **Hospital14** | **T635-2** | 5 | 0 | 1 | 0 |

**Supplementary Table 4. SNP analysis of CRAB isolates in clade2**

| **Hospital** | **Strains** | **R13** | **R44** | **R45** | **T44** | **T45** | **T54** | **R140** | **T140** |
| --- | --- | --- | --- | --- | --- | --- | --- | --- | --- |
| **Hospital 4** | **R13** | 0 | 52 | 51 | 51 | 34 | 54 | 63 | 62 |
| **Hospital 5** | **R44** | 52 | 0 | 35 | 35 | 18 | 37 | 61 | 60 |
| **Hospital 5** | **R45** | 51 | 35 | 0 | 1 | 17 | 3 | 27 | 28 |
| **Hospital 5** | **T44** | 51 | 35 | 1 | 0 | 18 | 2 | 26 | 27 |
| **Hospital 5** | **T45** | 34 | 18 | 17 | 18 | 0 | 20 | 45 | 45 |
| **Hospital 5** | **T54** | 54 | 37 | 3 | 2 | 20 | 0 | 27 | 33 |
| **Hospital 51** | **R140** | 63 | 61 | 27 | 26 | 45 | 27 | 0 | 6 |
| **Hospital 51** | **T140** | 62 | 60 | 28 | 27 | 45 | 33 | 6 | 0 |

**Supplementary Table 5. SNP analysis of CRAB isolates in clade3**

| **Hospital** | **Strains** | **R331** | **R338-1** | **R342** | **T338** | **T342** |
| --- | --- | --- | --- | --- | --- | --- |
| **Hospital 53** | **R331** | 0 | 2 | 4 | 2 | 3 |
| **Hospital 53** | **R338-1** | 2 | 0 | 0 | 1 | 0 |
| **Hospital 53** | **R342** | 4 | 0 | 0 | 1 | 0 |
| **Hospital 53** | **T338** | 2 | 1 | 1 | 0 | 2 |
| **Hospital 53** | **T342** | 3 | 0 | 0 | 2 | 0 |

**Supplementary Table 6. SNP analysis of CRAB isolates in clade4**

| **Hospital** | **Strain** | **R328** | **T330** | **R352** | **R354-1** | **R357-2** | **R359** | **T57** | **T59** | **R61** | **T61** | **T65** | **T66** | **T68-1** | **R72** | **T73-2** | **R139** | **R143-2** |
| --- | --- | --- | --- | --- | --- | --- | --- | --- | --- | --- | --- | --- | --- | --- | --- | --- | --- | --- |
| **Hospital 4** | **R328** | 0 | 3 | 15 | 25 | 19 | 20 | 54 | 25 | 53 | 50 | 65 | 53 | 56 | 53 | 27 | 30 | 28 |
| **Hospital 4** | **T330** | 3 | 0 | 18 | 21 | 20 | 19 | 50 | 24 | 49 | 50 | 61 | 51 | 49 | 51 | 25 | 26 | 25 |
| **Hospital 4** | **R352** | 15 | 18 | 0 | 19 | 14 | 15 | 56 | 28 | 55 | 51 | 65 | 56 | 57 | 55 | 31 | 38 | 37 |
| **Hospital 4** | **R354-1** | 25 | 21 | 19 | 0 | 6 | 6 | 62 | 30 | 54 | 57 | 64 | 55 | 53 | 56 | 29 | 40 | 35 |
| **Hospital 4** | **R357-2** | 19 | 20 | 14 | 6 | 0 | 4 | 64 | 29 | 56 | 59 | 67 | 58 | 57 | 55 | 31 | 40 | 35 |
| **Hospital 4** | **R359** | 20 | 19 | 15 | 6 | 4 | 0 | 65 | 28 | 57 | 57 | 68 | 61 | 56 | 60 | 27 | 42 | 37 |
| **Hospital 13** | **T57** | 54 | 50 | 56 | 62 | 64 | 65 | 0 | 60 | 0 | 3 | 19 | 3 | 8 | 4 | 60 | 64 | 63 |
| **Hospital 13** | **T59** | 25 | 24 | 28 | 30 | 29 | 28 | 60 | 0 | 58 | 56 | 70 | 62 | 57 | 59 | 4 | 45 | 43 |
| **Hospital 13** | **R61** | 53 | 49 | 55 | 54 | 56 | 57 | 0 | 58 | 0 | 2 | 16 | 1 | 7 | 3 | 58 | 63 | 62 |
| **Hospital 13** | **T61** | 50 | 50 | 51 | 57 | 59 | 57 | 3 | 56 | 2 | 0 | 18 | 3 | 9 | 5 | 57 | 63 | 63 |
| **Hospital 13** | **T65** | 65 | 61 | 65 | 64 | 67 | 68 | 19 | 70 | 16 | 18 | 0 | 17 | 19 | 20 | 68 | 60 | 72 |
| **Hospital 13** | **T66** | 53 | 51 | 56 | 55 | 58 | 61 | 3 | 62 | 1 | 3 | 17 | 0 | 9 | 2 | 63 | 65 | 64 |
| **Hospital 13** | **T68-1** | 56 | 49 | 57 | 53 | 57 | 56 | 8 | 57 | 7 | 9 | 19 | 9 | 0 | 12 | 57 | 62 | 61 |
| **Hospital 13** | **R72** | 53 | 51 | 55 | 56 | 55 | 60 | 4 | 59 | 3 | 5 | 20 | 2 | 12 | 0 | 60 | 63 | 64 |
| **Hospital 13** | **T73-2** | 27 | 25 | 31 | 29 | 31 | 27 | 60 | 4 | 58 | 57 | 68 | 63 | 57 | 60 | 0 | 46 | 45 |
| **Hospital 51** | **R139** | 30 | 26 | 38 | 40 | 40 | 42 | 64 | 45 | 63 | 63 | 60 | 65 | 62 | 63 | 46 | 0 | 2 |
| **Hospital 51** | **R143-2** | 28 | 25 | 37 | 35 | 35 | 37 | 63 | 43 | 62 | 63 | 72 | 64 | 61 | 64 | 45 | 2 | 0 |

**Supplementary Table 7. SNP analysis of CRAB isolates in clade5**

| **Hospital** | **Strain** | **R635-1** | **R638-2** |
| --- | --- | --- | --- |
| **Hospital 14** | **R635-1** | 0 | 62 |
| **Hospital 14** | **R638-2** | 62 | 0 |

**Supplementary Table 8. SNP analysis of CRAB isolates in clade6**

| **Hospital** | **Strain** | **R4-1** | **R6-1** | **T301-1** |
| --- | --- | --- | --- | --- |
| **Hospital 37** | **R4-1** | 0 | 10 | 19 |
| **Hospital 37** | **R6-1** | 10 | 0 | 29 |
| **Hospital 37** | **T301-1** | 19 | 29 | 0 |

**Supplementary Table 9. SNP analysis of CRAB isolates in clade7**

| **Hospital** | **Strain** | **R897-1** | **R902-2** | **T903-2** |
| --- | --- | --- | --- | --- |
| **Hospital 47** | **R897-1** | 0 | 9 | 24 |
| **Hospital 47** | **R902-2** | 9 | 0 | 16 |
| **Hospital 47** | **T903-2** | 24 | 16 | 0 |

**Supplementary Table10. SNP analysis of CRAB isolates in clade8**

| **Hospital** | **Strain** | **R476** | **T469** |
| --- | --- | --- | --- |
| **Hospital 31** | **R476** | 0 | 18 |
| **Hospital 31** | **T469** | 18 | 0 |

**Supplementary Table 11. SNP analysis of CRAB isolates in clade9**

| **Hospital** | **Strain** | **R821-1** | **R896-3** | **T896-2** |
| --- | --- | --- | --- | --- |
| **Hospital 47** | **R821-1** | 0 | 41 | 46 |
| **Hospital 47** | **R896-3** | 41 | 0 | 16 |
| **Hospital 47** | **T896-2** | 46 | 16 | 0 |

**Supplementary Table 12. SNP analysis of CRAB isolates in clade10**

| **Hospital** | **Strain** | **R28** | **R32** | **R40** | **T34** | **T35** | **T41** |
| --- | --- | --- | --- | --- | --- | --- | --- |
| **Hospital 39** | **R28** | 0 | 28 | 6 | 2 | 16 | 40 |
| **Hospital 39** | **R32** | 28 | 0 | 30 | 49 | 17 | 27 |
| **Hospital 39** | **R40** | 6 | 30 | 0 | 4 | 18 | 42 |
| **Hospital 39** | **T34** | 2 | 49 | 4 | 0 | 16 | 41 |
| **Hospital 39** | **T35** | 16 | 17 | 18 | 16 | 0 | 30 |
| **Hospital 39** | **T41** | 40 | 27 | 42 | 41 | 30 | 0 |

**Supplementary Table 13. SNP analysis of CRAB isolates in clade11**

| **Hospital** | **Strain** | **R914** | **R955** | **R960** | **T908** | **T912** |
| --- | --- | --- | --- | --- | --- | --- |
| **Hospital 28** | **R914** | 0 | 24 | 21 | 11 | 22 |
| **Hospital 28** | **R955** | 24 | 0 | 41 | 16 | 19 |
| **Hospital 28** | **R960** | 21 | 41 | 0 | 23 | 29 |
| **Hospital 28** | **T908** | 11 | 16 | 23 | 0 | 15 |
| **Hospital 28** | **T912** | 22 | 19 | 29 | 15 | 0 |

**Supplementary Table 14. SNP analysis of CRAB isolates in clade12**

| **Hospital** | **Strain** | **T362-2** | **R7-1** | **R87** | **R113** | **R129** | **R142** | **T141** | **T142** |
| --- | --- | --- | --- | --- | --- | --- | --- | --- | --- |
| **Hospital 4** | **T362-2** | 0 | 71 | 76 | 80 | 95 | 95 | 56 | 96 |
| **Hospital 4** | **R7-1** | 71 | 0 | 28 | 45 | 46 | 47 | 6 | 33 |
| **Hospital 36** | **R87** | 76 | 28 | 0 | 66 | 50 | 49 | 16 | 57 |
| **Hospital 51** | **R113** | 80 | 45 | 66 | 0 | 19 | 20 | 30 | 13 |
| **Hospital 51** | **R129** | 95 | 46 | 50 | 19 | 0 | 1 | 49 | 2 |
| **Hospital 51** | **R142** | 95 | 47 | 49 | 20 | 1 | 0 | 31 | 2 |
| **Hospital 51** | **T141** | 56 | 6 | 16 | 30 | 49 | 31 | 0 | 29 |
| **Hospital 51** | **T142** | 96 | 33 | 57 | 13 | 2 | 2 | 29 | 0 |

**Supplementary Table 15. SNP analysis of CRAB isolates in clade13**

| **Hospital** | **Strain** | **R996-3** | **T994** | **T996** | **R144-1** | **R144-2** | **T144** | **58** | **T899-2** | **R899-1** | **R905-2** |
| --- | --- | --- | --- | --- | --- | --- | --- | --- | --- | --- | --- |
| **Hospital 12** | **R996-3** | 0 | 0 | 4 | 23 | 21 | 21 | 18 | 22 | 22 | 22 |
| **Hospital 12** | **T994** | 0 | 0 | 4 | 21 | 20 | 20 | 17 | 21 | 21 | 21 |
| **Hospital 12** | **T996** | 4 | 4 | 0 | 21 | 20 | 20 | 17 | 21 | 21 | 21 |
| **Hospital 42** | **R144-1** | 23 | 21 | 21 | 0 | 1 | 2 | 13 | 26 | 26 | 26 |
| **Hospital 42** | **R144-2** | 21 | 20 | 20 | 1 | 0 | 0 | 11 | 25 | 25 | 25 |
| **Hospital 42** | **T144** | 21 | 20 | 20 | 2 | 0 | 0 | 11 | 26 | 25 | 25 |
| **Hospital 45** | **58** | 18 | 17 | 17 | 13 | 11 | 11 | 0 | 22 | 23 | 23 |
| **Hospital 47** | **T899-2** | 22 | 21 | 21 | 26 | 25 | 26 | 22 | 0 | 0 | 0 |
| **Hospital 47** | **R899-1** | 22 | 21 | 21 | 26 | 25 | 25 | 23 | 0 | 0 | 0 |
| **Hospital 47** | **R905-2** | 22 | 21 | 21 | 26 | 25 | 25 | 23 | 0 | 0 | 0 |

**Supplementary Table 16. SNP analysis of CRAB isolates in clade14**

| **Hospital** | **Strain** | **R350** | **R351-1** | **R361-1** | **R128-1** | **R128-2** | **T139** |
| --- | --- | --- | --- | --- | --- | --- | --- |
| **Hospital 4** | **R350** | 0 | 2 | 3 | 14 | 13 | 15 |
| **Hospital 4** | **R351-1** | 2 | 0 | 3 | 14 | 13 | 15 |
| **Hospital 4** | **R361-1** | 3 | 3 | 0 | 15 | 14 | 16 |
| **Hospital 51** | **R128-1** | 14 | 14 | 15 | 0 | 0 | 2 |
| **Hospital 51** | **R128-2** | 13 | 13 | 14 | 0 | 0 | 1 |
| **Hospital 51** | **T139** | 15 | 15 | 16 | 2 | 1 | 0 |

**Supplementary Table 17. SNP analysis of CRAB isolates in clade15**

| **Hospital** | **Strain** | **R671** | **R676** | **R679-1** | **R681** | **T681-1** |
| --- | --- | --- | --- | --- | --- | --- |
| **Hospital 7** | **R671** | 0 | 26 | 21 | 19 | 20 |
| **Hospital 7** | **R676** | 26 | 0 | 11 | 10 | 10 |
| **Hospital 7** | **R679-1** | 21 | 11 | 0 | 3 | 3 |
| **Hospital 7** | **R681** | 19 | 10 | 3 | 0 | 0 |
| **Hospital 7** | **T681-1** | 20 | 10 | 3 | 0 | 0 |

**Supplementary Table 18. SNP analysis of CRAB isolates in clade16**

| **Hospital** | **Strain** | **R994-1** | **R865-1** | **T865-1** | **R870** | **R871** | **R872-1** | **T872** | **R920** |
| --- | --- | --- | --- | --- | --- | --- | --- | --- | --- |
| **Hospital 12** | **R994-1** | 0 | 26 | 27 | 7 | 20 | 7 | 7 | 26 |
| **Hospital 19** | **R865-1** | 26 | 0 | 1 | 16 | 16 | 17 | 16 | 45 |
| **Hospital 19** | **T865-1** | 27 | 1 | 0 | 17 | 17 | 17 | 17 | 46 |
| **Hospital 19** | **R870** | 7 | 16 | 17 | 0 | 2 | 0 | 2 | 39 |
| **Hospital 19** | **R871** | 20 | 16 | 17 | 2 | 0 | 2 | 0 | 39 |
| **Hospital 19** | **R872-1** | 7 | 17 | 17 | 0 | 2 | 0 | 2 | 40 |
| **Hospital 19** | **T872** | 7 | 16 | 17 | 2 | 0 | 2 | 0 | 39 |
| **Hospital 25** | **R920** | 26 | 45 | 46 | 39 | 39 | 40 | 39 | 0 |

**Supplementary Table 19. SNP analysis of CRAB isolates in clade17**

| **Hospital** | **Strain** | **T656-2** | **R666** | **T666** | **R670-2** | **T670** |
| --- | --- | --- | --- | --- | --- | --- |
| **Hospital 33** | **T656-2** | 0 | 8 | 9 | 10 | 12 |
| **Hospital 33** | **R666** | 8 | 0 | 1 | 20 | 2 |
| **Hospital 33** | **T666** | 9 | 1 | 0 | 2 | 5 |
| **Hospital 33** | **R670-2** | 10 | 20 | 2 | 0 | 1 |
| **Hospital 33** | **T670** | 12 | 2 | 5 | 1 | 0 |

**Supplementary Table 20. SNP analysis of CRAB isolates in clade18**

| **Hospital** | **Strain** | **R735** | **R34** | **R312-2** | **R166** | **R181** | **R599-1** | **R602-1** | **R604-1** | **R606-1** | **R617-2** | **R625** | **T182** | **T599-2** | **T625-2** |
| --- | --- | --- | --- | --- | --- | --- | --- | --- | --- | --- | --- | --- | --- | --- | --- |
| **Hospital 18** | **R735** | 0 | 185 | 88 | 58 | 75 | 68 | 83 | 61 | 57 | 64 | 65 | 59 | 62 | 70 |
| **Hospital 29** | **R34** | 185 | 0 | 86 | 87 | 77 | 110 | 93 | 73 | 83 | 103 | 105 | 110 | 102 | 112 |
| **Hospital 37** | **R312-2** | 88 | 86 | 0 | 39 | 45 | 64 | 58 | 39 | 41 | 61 | 61 | 62 | 59 | 66 |
| **Hospital 41** | **R166** | 58 | 87 | 39 | 0 | 23 | 6 | 27 | 6 | 5 | 9 | 11 | 5 | 5 | 12 |
| **Hospital 41** | **R181** | 75 | 77 | 45 | 23 | 0 | 29 | 5 | 21 | 20 | 18 | 26 | 21 | 23 | 32 |
| **Hospital 41** | **R599-1** | 68 | 110 | 64 | 6 | 29 | 0 | 31 | 14 | 8 | 12 | 14 | 5 | 8 | 15 |
| **Hospital 41** | **R602-1** | 83 | 93 | 58 | 27 | 5 | 31 | 0 | 25 | 25 | 27 | 34 | 28 | 29 | 33 |
| **Hospital 41** | **R604-1** | 61 | 73 | 39 | 6 | 21 | 14 | 25 | 0 | 1 | 5 | 8 | 6 | 7 | 15 |
| **Hospital 41** | **R606-1** | 57 | 83 | 41 | 5 | 20 | 8 | 25 | 1 | 0 | 7 | 10 | 5 | 7 | 9 |
| **Hospital 41** | **R617-2** | 64 | 103 | 61 | 9 | 18 | 12 | 27 | 5 | 7 | 0 | 15 | 8 | 8 | 13 |
| **Hospital 41** | **R625** | 65 | 105 | 61 | 11 | 26 | 14 | 34 | 8 | 10 | 15 | 0 | 11 | 14 | 2 |
| **Hospital 41** | **T182** | 59 | 110 | 62 | 5 | 21 | 5 | 28 | 6 | 5 | 8 | 11 | 0 | 5 | 12 |
| **Hospital 41** | **T599-2** | 62 | 102 | 59 | 5 | 23 | 8 | 29 | 7 | 7 | 8 | 14 | 5 | 0 | 14 |
| **Hospital 41** | **T625-2** | 70 | 112 | 66 | 12 | 32 | 15 | 33 | 15 | 9 | 13 | 2 | 12 | 14 | 0 |

**Supplementary Table 21. SNP analysis of CRAB isolates in clade19**

| **Hospital** | **Strain** | **T987** | **T984-1** |
| --- | --- | --- | --- |
| **Hospital 12** | **T987** | 0 | 1 |
| **Hospital 54** | **T984-1** | 1 | 0 |

**Supplementary Table 22. SNP analysis of CRAB isolates in clade20**

| **Hospital** | **Strain** | **T1007** | **T316-1** |
| --- | --- | --- | --- |
| **Hospital 1** | **T1007** | 0 | 12 |
| **Hospital 37** | **T316-1** | 12 | 0 |

**Supplementary Table 23. SNP analysis of CRAB isolates in clade21**

| **Hospital** | **Strain** | **R577** | **R581** | **R583** | **T575** | **T577** | **T582** | **T583** |
| --- | --- | --- | --- | --- | --- | --- | --- | --- |
| **hosptial 22** | **R577** | 0 | 6 | 5 | 3 | 3 | 3 | 5 |
| **hosptial 22** | **R581** | 6 | 0 | 9 | 9 | 8 | 8 | 11 |
| **hosptial 22** | **R583** | 5 | 9 | 0 | 3 | 4 | 4 | 5 |
| **hosptial 22** | **T575** | 3 | 9 | 3 | 0 | 1 | 4 | 7 |
| **hosptial 22** | **T577** | 3 | 8 | 4 | 1 | 0 | 4 | 7 |
| **hosptial 22** | **T582** | 3 | 8 | 4 | 4 | 4 | 0 | 9 |
| **hosptial 22** | **T583** | 5 | 11 | 5 | 7 | 7 | 9 | 0 |

**Supplementary Table 24. SNP analysis of CRAB isolates in clade22**

| **Hospital** | **Provinces** | **Strain** | **R365-1** | **R699** | **R700** | **R539** | **R547-1** | **R551-1** | **R940** | **T940** | **R991** | **T991** | **R753** | **R848** | **T848-1** | **R850** | **R477** | **R16** | **T16** | **TJ1-2** | **R898-2** | **T898** | **T111-2** |
| --- | --- | --- | --- | --- | --- | --- | --- | --- | --- | --- | --- | --- | --- | --- | --- | --- | --- | --- | --- | --- | --- | --- | --- |
| **Hospital 6** | **Zhejiang** | **R365-1** | 0 | 78 | 78 | 70 | 70 | 106 | 80 | 76 | 78 | 78 | 46 | 83 | 82 | 83 | 50 | 63 | 63 | 85 | 73 | 59 | 76 |
| **Hospital 9** | **Shanxi** | **R699** | 78 | 0 | 3 | 69 | 66 | 96 | 62 | 61 | 81 | 82 | 35 | 77 | 76 | 77 | 52 | 52 | 52 | 83 | 79 | 59 | 66 |
| **Hospital 9** | **Shanxi** | **R700** | 78 | 3 | 0 | 68 | 65 | 99 | 59 | 56 | 81 | 81 | 34 | 75 | 75 | 76 | 51 | 50 | 53 | 81 | 80 | 58 | 66 |
| **Hospital 10** | **Fujian** | **R539** | 70 | 69 | 68 | 0 | 0 | 49 | 68 | 70 | 53 | 55 | 44 | 87 | 87 | 87 | 51 | 54 | 70 | 69 | 51 | 50 | 54 |
| **Hospital 10** | **Fujian** | **R547-1** | 70 | 66 | 65 | 0 | 0 | 49 | 72 | 69 | 49 | 52 | 42 | 83 | 83 | 83 | 49 | 51 | 70 | 71 | 53 | 52 | 50 |
| **Hospital 10** | **Fujian** | **R551-1** | 106 | 96 | 99 | 49 | 49 | 0 | 129 | 127 | 83 | 95 | 93 | 141 | 141 | 143 | 87 | 94 | 95 | 128 | 92 | 92 | 93 |
| **Hospital 11** | **Guangxi** | **R940** | 80 | 62 | 59 | 68 | 72 | 129 | 0 | 1 | 47 | 50 | 111 | 87 | 85 | 106 | 62 | 73 | 73 | 2 | 40 | 42 | 97 |
| **Hospital 11** | **Guangxi** | **T940** | 76 | 61 | 56 | 70 | 69 | 127 | 1 | 0 | 50 | 49 | 108 | 89 | 86 | 109 | 55 | 72 | 70 | 4 | 41 | 43 | 98 |
| **Hospital 12** | **Hainan** | **R991** | 78 | 81 | 81 | 53 | 49 | 83 | 47 | 50 | 0 | 17 | 55 | 78 | 78 | 79 | 46 | 69 | 72 | 55 | 49 | 46 | 80 |
| **Hospital 12** | **Hainan** | **T991** | 78 | 82 | 81 | 55 | 52 | 95 | 50 | 49 | 17 | 0 | 55 | 81 | 80 | 79 | 44 | 70 | 71 | 58 | 51 | 48 | 81 |
| **Hospital 17** | **Henan** | **R753** | 46 | 35 | 34 | 44 | 42 | 93 | 111 | 108 | 55 | 55 | 0 | 70 | 69 | 68 | 47 | 37 | 41 | 112 | 50 | 51 | 34 |
| **Hospital 23** | **Liaoning** | **R848** | 83 | 77 | 75 | 87 | 83 | 141 | 87 | 89 | 78 | 81 | 70 | 0 | 0 | 10 | 74 | 82 | 84 | 87 | 72 | 73 | 101 |
| **Hospital 23** | **Liaoning** | **T848-1** | 82 | 76 | 75 | 87 | 83 | 141 | 85 | 86 | 78 | 80 | 69 | 0 | 0 | 7 | 69 | 82 | 83 | 86 | 72 | 73 | 100 |
| **Hospital 23** | **Liaoning** | **R850** | 83 | 77 | 76 | 87 | 83 | 143 | 106 | 109 | 79 | 79 | 68 | 10 | 7 | 0 | 73 | 82 | 84 | 108 | 73 | 74 | 100 |
| **Hospital 31** | **Jiangxi** | **R477** | 50 | 52 | 51 | 51 | 49 | 87 | 62 | 55 | 46 | 44 | 47 | 74 | 69 | 73 | 0 | 58 | 57 | 59 | 42 | 40 | 77 |
| **Hospital 38** | **Zhejiang** | **R16** | 63 | 52 | 50 | 54 | 51 | 94 | 73 | 72 | 69 | 70 | 37 | 82 | 82 | 82 | 58 | 0 | 5 | 72 | 62 | 61 | 45 |
| **Hospital 38** | **Zhejiang** | **T16** | 63 | 52 | 53 | 70 | 70 | 95 | 73 | 70 | 72 | 71 | 41 | 84 | 83 | 84 | 57 | 5 | 0 | 72 | 66 | 65 | 61 |
| **Hospital 44** | **Hunan** | **TJ1-2** | 85 | 83 | 81 | 69 | 71 | 128 | 2 | 4 | 55 | 58 | 112 | 87 | 86 | 108 | 59 | 72 | 72 | 0 | 49 | 41 | 98 |
| **Hospital 47** | **Hunan** | **R898-2** | 73 | 79 | 80 | 51 | 53 | 92 | 40 | 41 | 49 | 51 | 50 | 72 | 72 | 73 | 42 | 62 | 66 | 49 | 0 | 0 | 77 |
| **Hospital 47** | **Hunan** | **T898** | 59 | 59 | 58 | 50 | 52 | 92 | 42 | 43 | 46 | 48 | 51 | 73 | 73 | 74 | 40 | 61 | 65 | 41 | 0 | 0 | 76 |
| **Hospital 51** | **Zhejiang** | **T111-2** | 76 | 66 | 66 | 54 | 50 | 93 | 97 | 98 | 80 | 81 | 34 | 101 | 100 | 100 | 77 | 45 | 61 | 98 | 77 | 76 | 0 |

**Supplementary Table 25. SNP analysis of representative strains from 22 clonal clades**

| **Hospital** | **Strain** | **R632**  **-2** | **R13** | **R331** | **R328** | **R635**  **-1** | **R821**  **-1** | **R471** | **R897**  **-1** | **R4-1** | **R28** | **R914** | **T362**  **-2** | **R996**  **-3** | **R350** | **R671** | **R994**  **-1** | **T656**  **-2** | **R735** | **T987** | **T1007** | **R577** | **R365-1** |
| --- | --- | --- | --- | --- | --- | --- | --- | --- | --- | --- | --- | --- | --- | --- | --- | --- | --- | --- | --- | --- | --- | --- | --- |
| **Clade1** | **R632-2** | 0 | 1786 | 1944 | 1456 | 6186 | 4100 | 57085 | 5845 | 5798 | 2950 | 4911 | 5204 | 1537 | 1453 | 1948 | 1421 | 2017 | 2330 | 2538 | 2985 | 2300 | 2251 |
| **Clade2** | **R13** | 1786 | 0 | 1439 | 1200 | 5942 | 3544 | 56633 | 5420 | 5055 | 2441 | 4462 | 4814 | 920 | 996 | 1453 | 986 | 1706 | 2088 | 2151 | 2591 | 1909 | 1848 |
| **Clade3** | **R331** | 1944 | 1439 | 0 | 759 | 5848 | 3870 | 56732 | 6108 | 5867 | 2706 | 4970 | 4905 | 985 | 1202 | 1740 | 1262 | 1917 | 1944 | 2632 | 3001 | 2124 | 2107 |
| **Clade4** | **R328** | 1456 | 1200 | 759 | 0 | 5157 | 2942 | 56771 | 5727 | 5366 | 2017 | 4508 | 4797 | 890 | 888 | 1427 | 874 | 1568 | 1919 | 2033 | 2462 | 1753 | 1717 |
| **Clade5** | **R635-1** | 6186 | 5942 | 5848 | 5157 | 0 | 5653 | 57260 | 7815 | 7430 | 6076 | 6451 | 6764 | 5965 | 5947 | 6418 | 5746 | 6456 | 6562 | 6630 | 6956 | 6385 | 6402 |
| **Clade6** | **R821-1** | 4100 | 3544 | 3870 | 2942 | 5653 | 0 | 56978 | 5421 | 5047 | 3632 | 5209 | 5585 | 3593 | 3597 | 4182 | 3306 | 3792 | 3239 | 4218 | 4540 | 4007 | 4016 |
| **Clade7** | **R471** | 57085 | 56633 | 56732 | 56771 | 57260 | 56978 | 0 | 55622 | 55767 | 57180 | 57632 | 57070 | 56960 | 56512 | 56714 | 56837 | 56060 | 56723 | 56565 | 56622 | 56664 | 57004 |
| **Clade8** | **R897-1** | 5845 | 5420 | 6108 | 5727 | 7815 | 5421 | 55622 | 0 | 1122 | 5959 | 7076 | 7347 | 5570 | 5547 | 6098 | 5084 | 5061 | 4859 | 5074 | 4866 | 5344 | 5429 |
| **Clade9** | **R4-1** | 5798 | 5055 | 5867 | 5366 | 7430 | 5047 | 55767 | 1122 | 0 | 5577 | 6673 | 6929 | 5188 | 5174 | 5649 | 4417 | 5061 | 4354 | 4011 | 3791 | 4311 | 4386 |
| **Clade10** | **R28** | 2950 | 2441 | 2706 | 2017 | 6076 | 3632 | 57180 | 5959 | 5577 | 0 | 2664 | 2939 | 2204 | 2154 | 2639 | 2141 | 2861 | 3197 | 3288 | 3590 | 3048 | 2997 |
| **Clade11** | **R914** | 4911 | 4462 | 4970 | 4508 | 6451 | 5209 | 57632 | 7076 | 6673 | 2664 | 0 | 488 | 4249 | 4326 | 4933 | 4298 | 5042 | 5434 | 5446 | 5711 | 5253 | 5227 |
| **Clade12** | **T362-2** | 5204 | 4814 | 4905 | 4797 | 6764 | 5585 | 57070 | 7347 | 6929 | 2939 | 488 | 0 | 4606 | 4603 | 5017 | 4659 | 5356 | 5644 | 5753 | 6010 | 5498 | 5466 |
| **Clade13** | **R996-3** | 1537 | 920 | 985 | 890 | 5965 | 3593 | 56960 | 5570 | 5188 | 2204 | 4249 | 4606 | 0 | 443 | 1019 | 472 | 986 | 1337 | 1624 | 2068 | 1197 | 1329 |
| **Clade14** | **R350** | 1453 | 996 | 1202 | 888 | 5947 | 3597 | 56512 | 5547 | 5174 | 2154 | 4326 | 4603 | 443 | 0 | 609 | 139 | 1103 | 1449 | 1274 | 1897 | 1229 | 976 |
| **Clade15** | **R671** | 1948 | 1453 | 1740 | 1427 | 6418 | 4182 | 56714 | 6098 | 5649 | 2639 | 4933 | 5017 | 1019 | 609 | 0 | 417 | 1449 | 1829 | 1865 | 2411 | 1614 | 1445 |
| **Clade16** | **R994-1** | 1421 | 986 | 1262 | 874 | 5746 | 3306 | 56837 | 5084 | 4417 | 2141 | 4298 | 4659 | 472 | 139 | 417 | 0 | 636 | 956 | 446 | 1046 | 346 | 132 |
| **Clade17** | **T656-2** | 2017 | 1706 | 1917 | 1568 | 6456 | 3792 | 56060 | 5061 | 5061 | 2861 | 5042 | 5356 | 986 | 1103 | 1449 | 636 | 0 | 985 | 1466 | 1990 | 843 | 1029 |
| **Clade18** | **R735** | 2330 | 2088 | 1944 | 1919 | 6562 | 3239 | 56723 | 4859 | 4354 | 3197 | 5434 | 5644 | 1337 | 1449 | 1829 | 956 | 985 | 0 | 1220 | 1710 | 629 | 844 |
| **Clade19** | **T987** | 2538 | 2151 | 2632 | 2033 | 6630 | 4218 | 56565 | 5074 | 4011 | 3288 | 5446 | 5753 | 1624 | 1274 | 1865 | 446 | 1466 | 1220 | 0 | 640 | 647 | 469 |
| **Clade20** | **T1007** | 2985 | 2591 | 3001 | 2462 | 6956 | 4540 | 56622 | 4866 | 3791 | 3590 | 5711 | 6010 | 2068 | 1897 | 2411 | 1046 | 1990 | 1710 | 640 | 0 | 1221 | 1054 |
| **Clade21** | **R577** | 2300 | 1909 | 2124 | 1753 | 6385 | 4007 | 56664 | 5344 | 4311 | 3048 | 5253 | 5498 | 1197 | 1229 | 1614 | 346 | 843 | 629 | 647 | 1221 | 0 | 234 |
| **Clade22** | **R365-1** | 2251 | 1848 | 2107 | 1717 | 6402 | 4016 | 57004 | 5429 | 4386 | 2997 | 5227 | 5466 | 1329 | 976 | 1445 | 132 | 1029 | 844 | 469 | 1054 | 234 | 0 |


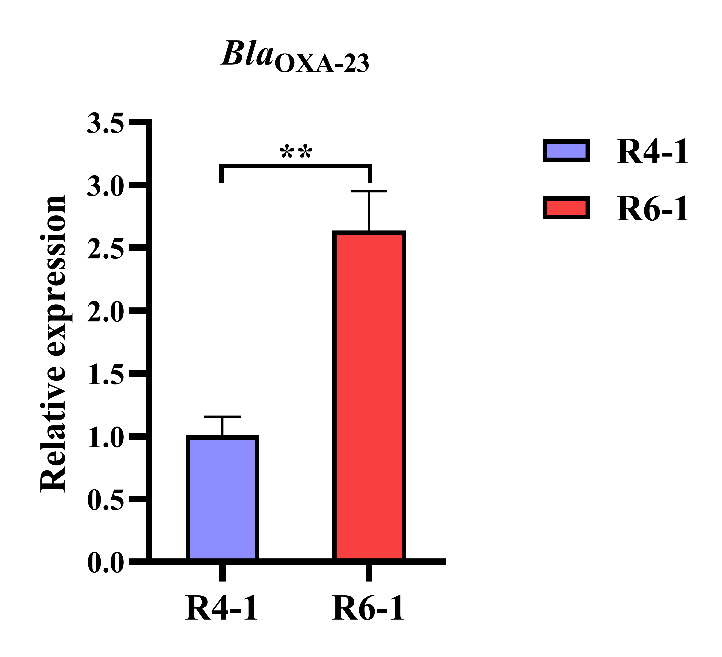


**Supplementary Figure S1. Relative expression levels of *bla*_OXA-23_ in strains R4-1 and R6-1.** Statistically significant difference was observed between the relative expression of *bla*_OXA-23_ gene in R4-1 (CAV-susceptible) and R6-1(CAV-resistant).


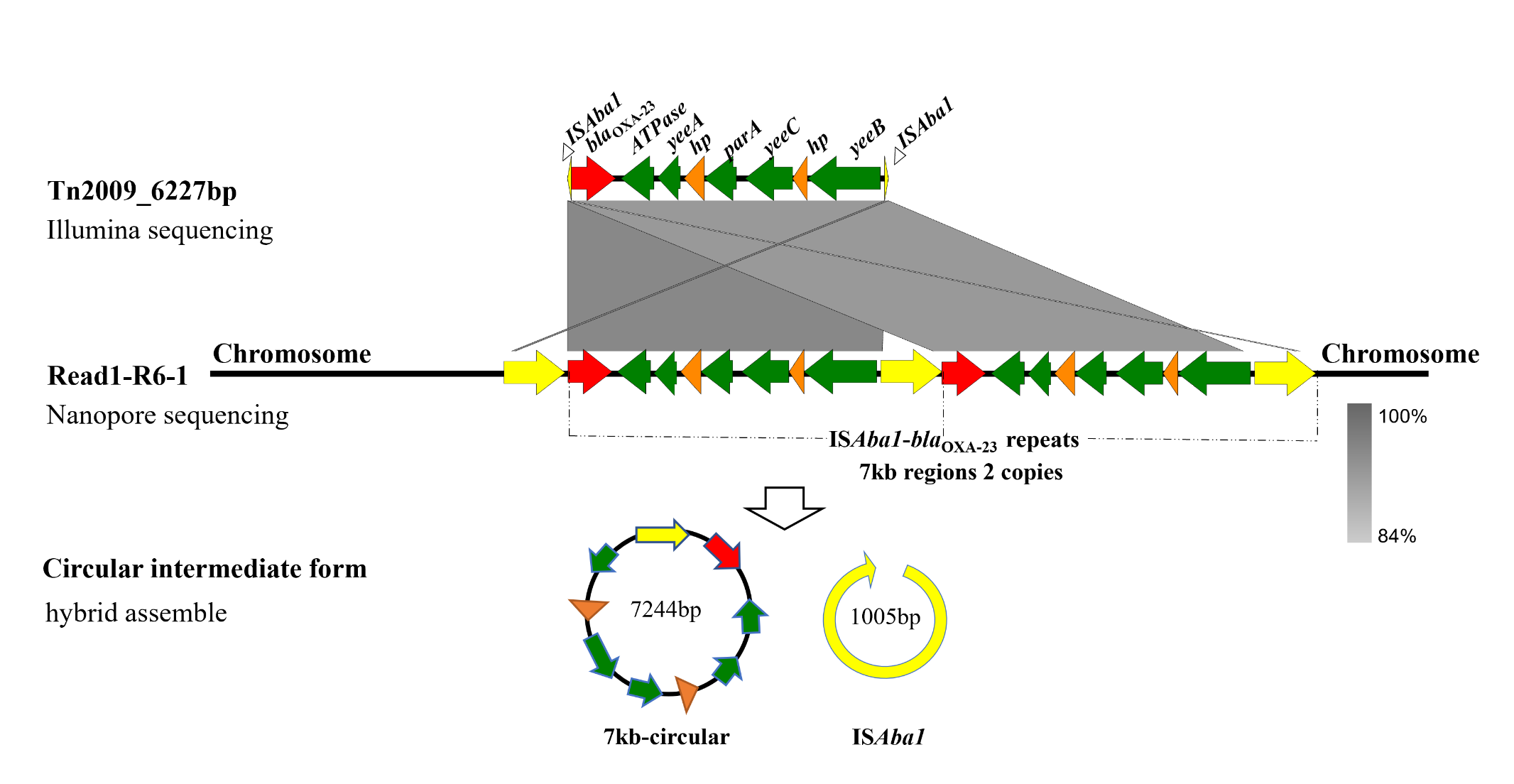


**Supplementary Figure S2. Formation of a circular intermediate by multiplication of *bla*_OXA-23_ in the chromosome.** Alignment between genetic structures of Tn2009 and relevant nanopore raw reads, suggesting that two copies of *bla*_OXA-23_ were tandemly clustered into the chromosome of R6-1, indicating that multiple copies of *bla*_OXA-23_-bearing structure may form circular intermediates and enhance the expression level of *bla*_OXA-23._
